# The Effect of Second-Victim-Related Distress and Support on Work-Related Outcomes in Tertiary Care Hospitals in Kelantan, Malaysia

**DOI:** 10.3390/ijerph19116454

**Published:** 2022-05-26

**Authors:** Ahmad Zulfahmi Mohd Kamaruzaman, Mohd Ismail Ibrahim, Ariffin Marzuki Mokhtar, Maizun Mohd Zain, Saiful Nazri Satiman, Najib Majdi Yaacob

**Affiliations:** 1 Department of Community Medicine, School of Medical Sciences, Universiti Sains Malaysia, Kubang Kerian, Kota Bharu 16150, Kelantan, Malaysia; drzulfahmi@student.usm.my; 2 Department of Anesthesiology and Intensive Care, School of Medical Sciences, Universiti Sains Malaysia, Kubang Kerian, Kota Bharu 16150, Kelantan, Malaysia; ariffinm@usm.my; 3 Public Health Unit, Hospital Raja Perempuan Zainab II, Kota Bharu 16150, Kelantan, Malaysia; drmaizun@moh.gov.my; 4 Medical Division, Kelantan State Health Department, Kota Bharu 16150, Kelantan, Malaysia; drsaifulnazri@moh.gov.my; 5 Unit of Biostatistics and Research Methodology, School of Medical Sciences, Universiti Sains Malaysia, Kubang Kerian, Kota Bharu 16150, Kelantan, Malaysia; najibmy@usm.my

**Keywords:** second victims, patient safety incidents, hierarchical linear regression, mediation, support

## Abstract

After a patient safety incident, the involved healthcare providers may experience sustained second-victim distress and reduced professional efficacy, with subsequent negative work-related outcomes and the cultivation of resilience. This study aims to investigate the factors affecting negative work-related outcomes and resilience with a hypothetical triad of support as the mediators: colleague, supervisor, and institutional support. This cross-sectional study recruited 733 healthcare providers from three tertiary care hospitals in Kelantan, Malaysia. Three steps of hierarchical linear regression were developed for both outcomes (negative work-related outcomes and resilience). Four multiple mediator models of the support triad were analyzed. Second-victim distress, professional efficacy, and the support triad contributed significantly in all the regression models. Colleague support partially mediated the relationship defining the effects of professional efficacy on negative work-related outcomes and resilience, whereas colleague and supervisor support partially mediated the effects of second-victim distress on negative work-related outcomes. Similar results were found regarding resilience, with all support triads producing similar results. As mediators, the support triads ameliorated the effect of second-victim distress on negative work-related outcomes and resilience, suggesting an important role of having good support, especially after encountering patient safety incidents.

## 1. Introduction

Adverse events during clinical care are unwelcome, but unfortunately unavoidable. In the aftermath of any adverse event, the healthcare institution immediately focuses its attention on the affected patients and families—the first victims [1]. Indeed, witnessing and first-hand encountering patient harm is a difficult and traumatizing experience for healthcare providers (HCPs).

In a seminal publication of what have been termed ‘second victim’, Albert Wu explained that the compromised and often overlooked HCPs could be wounded emotionally (self-doubt, guilt, anxiety, anger, embarrassment, frustration, self-hatred, and depression) [2,3,4,5,6,7,8,9,10,11,12], cognitively (compassion dissatisfaction, burnout, secondary traumatic stress, and troubling memories) [5,13,14,15,16], and physically (sleeping disturbances, nausea, increase in blood pressure, and heart rate or respiratory rate) [1,3,17,18] as sequelae of adverse events. Later evidence suggests that second victims are not confined to adverse events, but to any kind of patient safety incident (PSI), event or circumstance that could have resulted, or did result, in unnecessary harm to a patient [19]. The continuum of PSI includes near-misses, incident reporting, morbidity and mortality settings, and any adverse event [20,21,22].

Furthermore, if left unattended, the second victims could further deteriorate and progress to reduced professional competence (increase turnover rate and absenteeism, practicing defensive medicine, repercussive clinical error, and sub-optimal patient care) [23,24,25,26,27,28,29,30,31], post-traumatic stress disorder, burnout, committing self-inflicted injury, or suicide [32,33,34]. The myriad of symptoms and complications are known as second-victim syndrome (SVS). Turnover intention, for instance, refers to the process of HCPs leaving an organization. A high turnover rate results in increased costs due to selection and recruitment. Furthermore, the productivity and clinical services provided by incoming new substitutes may not be on par with those of the experienced one who left [35]. This is similarly observed with absenteeism, the period taken to cope with and self-reflect after a PSI. Worse, starting from an individual concern, SVS can create a domino effect toward healthcare organizations—the third victims—by reputational, medico-legal, human resources, or monetary issues [36,37,38]. However, despite the negativity of SVS, more recent evidence has demonstrated that SVS could perhaps cultivate resilience, alleviate stress, and improve mental health for HCPs [39,40,41,42,43].

Globally, the occurrence of second victims has been recorded in many countries and healthcare settings. The captured incidence of second victims has portrayed a wide range of conclusions. In high-stake clinical disciplines, it can be as remarkable as 90% [9,44,45,46,47,48,49,50,51,52]. Decades ago, the popular approach in handling SVS was retributive justice or applying a punitive approach after any PSI, as recorded in the earliest ground-breaking second-victim studies in the 1980s. The culprits were then blamed and punished accordingly [53,54]. Since then, this approach has been largely considered unethical, harassing, intimidating, and denying the rights of the second victims. Although a conservative approach is still embraced, the paradigm has been slowly shifted toward an inculcating-only culture in healthcare organizations. Instead of finding fault and placing blame, the restorative justice approach proposes organizational support as one of the important elements for managing SVS [55,56,57].

The continuum of organizational support encompasses the triad of colleague, supervisor, and institutional support. After any PSI, colleagues usually act as first responders, as they are the closest to the second victims. Colleague or peer support is regarded as the most conducive, sought-after, and successful kind of support [3,13,27,58,59,60,61,62,63]. Besides colleague support, good supervisor support provides reference and professional affirmation for the second victims [3,61]. Taking into account the systemic perspective, the well-being of HCPs and conducive working environments are included as one of the elements in the highly regarded quadruple aim: a compass to optimize health system performance [64,65]. Therefore, in mitigating SVS, institutional or healthcare organizations also play a detrimental role. There are various types of support provided by the institution. These can range from wellness programs, pastoral care, employee assistance programs to organized peer support, and psychiatric care. In addition, the level of institutional support can be varied according to the respective organization. SVS mitigation and intervention programs have been put forward, as exemplified by Medically Induced Trauma Support Services, John Hopkins Hospital: Resilience in Stressful Events (RISE), University of Missouri Health System: for FORYOU team, Institute for Healthcare Improvement: Building A Clinical Support Program, and others [60,66,67,68,69].

Unlike the extensive literature from Western countries, no studies in Malaysia have explored the SVS and its management plan. Thus, this study aims to examine the relationship between second-victim-related distress (second-victim distress and professional efficacy) and two outcomes: negative work-related outcomes (e.g., absenteeism, presenteeism, turnover, and patient safety incidents) and resilience. The organizational support offered was measured simultaneously and hypothesized as the potential third variable that mediated the relationship between second-victim-related distress and the two aforementioned outcomes.

## 2. Materials and Methods

### 2.1. Study Design and Participants

A cross-sectional study was conducted between September and December 2021 in three tertiary care hospitals in Kelantan, Malaysia. The selected respondents were HCPs (doctors, nurses, and assistant medical officers) who were routinely working in patient care, had previously encountered some form of PSI within the last 5 years, and declared not having any psychiatric illnesses. The recruitment process was regulated by hospital administrative officials and co-researchers. A list of total HCPs was pre-gathered, and using systematic random sampling, the respondents were then selected. The questionnaires were administered online and did not favor any direct meetings. The respondents had to first meet the inclusion criteria and consent to enrolment before being permitted to attempt the remaining parts of the questionnaires.

### 2.2. The M-SVEST-R Instrument

The Second Victim Experience and Support Tool (SVEST) is a tool used to measure SVS and its support. It has been widely translated and validated into many languages, such as Spanish (Spain and Argentina), Italian, Korean, Chinese, Persian (Iran), Danish, and German [70,71,72,73,74,75,76,77]. An improvised version of the SVEST—Revised Second Victim Experience and Support Tool (SVEST-R) was developed by Winning [39]. The SVEST-R was translated and validated into Malay, the national language of Malaysia, named the Malaysian version of the revised Second Victim Experience and Support Tool (M-SVEST-R), and deployed for this study [78].

The M-SVEST-R questionnaire consists of seven dimensions: second victim distress (psychological distress (four items) and physical distress (five items)), colleague support (three items), supervisor support (three items), institutional support (two items), professional self-efficacy (four items), negative work-related outcomes (turnover intentions (four items) and absenteeism (three items)), and resilience (four items). All items are close-ended questions using a five-point Likert scale ranging from 1 (strongly disagree) to 5 (strongly agree). High SVEST scores indicate a high prevalence of second-victim responses, a perception of insufficient support resources, and the magnitude of negative work-related outcomes and resilience.

The M-SVEST-R demonstrated good construct validity (chi-square test, χ^2^ = 797), degree of freedom (DOF) = 418, root-mean-square error of approximation (RMSEA) = 0.05, comparative fit index (CFI) = 0.946, and standardized root mean squared residual (SRMR) = 0.055. Factor loadings of all items ranged from 0.6 to 0.867, while Raykov’s rho ranged from 0.68 for colleague support to 0.93 for second-victim distress (total scale at 0.83).

### 2.3. Data Analysis

The data analysis was carried out using R software (R Core Team: Vienna, Austria, 2020) [79]. Descriptive statistics of sociodemographic variables, dimensions, and items were measured using mean (standard deviation) for numerical data and count (percentage) for categorical data. The percentage of agreement was also introduced according to the number of participants who achieved a mean score ≥ 4.0 (a proxy that shows a negative outcome for each dimension had occurred due to a second-victim experience) [22,70].

We used hierarchical linear regression to evaluate the contribution of predictors to the intended outcomes. The method was a sequential process involving the entry of predictor variables into blocks, based on a theoretical background [80]. As with standard multiple regression, hierarchical linear regression assumes an adequate sample size, avoids multicollinearity between predictors, removes outliers, and suggests normality, linearity, and homoscedasticity of residuals [81]. Upon agreement with the assumptions, the analysis continued with the block-by-block predictor insertion as follows:

i. Block 1—sociodemographic variables (age, gender, length of working experience, occupation, marital status, and last involvement in PSI).
ii. Block 2—second-victim distress and professional efficacy.
iii. Block 3—colleague, supervisor, and institutional support.

Each predictor was evaluated in terms of added prediction toward the outcome after the previous predictors had been controlled. The overall model and the relative contribution of each block were analyzed. The analysis started with the first outcome—negative work-related outcomes—and then repeated for the second outcome—resilience.

#### Mediation Analysis

After hierarchical linear regression, this study continued with a mediation analysis. To prove the mediator, Baron and Kenny’s criteria of predetermined conditions were fulfilled as follows:

1. The total effect of X on Y (c) must be significant.
2. The effect of X on M (a) must be significant.
3. The effect of M on Y, controlled for X (b), must be significant.
4. The effect of X on Y controlling for M (c′) should be zero.

If all conditions agree, it is full mediation. However, if all are fulfilled but the number 4 is not, it is considered partial mediation [82]. Partial mediation can then be categorized into complementary (the direct and indirect effect points in the same direction) and competitive (the direct and indirect effect points in different directions) [83].

In this study, incorporating Baron and Kenny’s conditions, multiple mediators were introduced simultaneously into the model (refer supplementary file). According to theory, there were three hypothetical mediators, colleague, supervisor, and institutional support, mediating the relationship between predictors (second-victim distress and professional efficacy) and outcomes (negative work-related outcomes and resilience). With such analysis, there were four directed acyclic graphs of multiple mediators to present.

After distinguishing the mediator effect using Baron and Kenny’s conditions to confirm the analysis, a computational resampling procedure, known as the bootstrapping technique, was generated. This produces a sampling dispersion for estimating the indirect effect, the direct effect, and the significant value [84,85,86]. The bootstrapping result was compared with the ordinary regression results to reaffirm mediation.

### 2.4. Sample Size Determination

Using G*Power 3.1 software (Department of General Psychology, Düsseldorf University, Germany, 2009) [87] on hierarchical linear regression, there were 344 respondents to obtain (inclusive of twenty percent of possible dropouts). However, the mediation analysis utilized Monte Carlo power analysis of indirect effects [88] and targeted 369 respondents after considering dropouts. 

### 2.5. Ethical Considerations

The study was approved by the Medical Research and Ethics Committee (MREC) of the Ministry of Health (NMRR-21-171-58022) and the Human Research and Ethics Committee of Universiti Sains Malaysia (JEPeM Code: USM/JEPeM/21020161). Data confidentiality was sternly preserved. Data access was restricted only to the authors and supervisors. Reporting and publication were conducted anonymously, excluding any personal identification.

## 3. Results

This study initially recruited a total of 765 HCPs, and 740 of them acknowledged that they had experienced at least an episode of PSI within the last 5 years. Out of these eligible participants, seven HCPs were unable to complete all the questionnaires or had some items missing the data. In total, 733 participants were considered in this study, which accounted for a 95.8% response rate.

The participants comprised 596 nurses (81.3%), 114 medical doctors (15.6%), and 23 assistant medical officers (3.1%), the majority of whom were women (630, 85.9%) and married (618, 84.3%). The mean age of the participants was 36.7 years, with 12 years of average working experience and 2 years of average time of last encounter with PSI.

According to the respective departments, 168 participants (22.3%) were from anesthesiology and intensive care, 130 participants (17.7%) were from internal medicine, 89 participants (12.1%) were from orthopedic, 87 participants (11.9%) were from obstetrics and gynecology, 79 participants (10.8%) were from surgery, 104 participants (14.2%) were from pediatric, 30 participants (4.1%) were from emergency and trauma, and 44 participants (6%) were from other departments (Table 1).

The mean scores ranged from 1.95 (SD: 0.87) for colleague support to 2.9 (SD: 1.1) for institutional support. As for percentage of agreement (SVEST scores), the values ranged from 1.6% (colleague support) to 12.1% (institutional support). These percentages reflected that 1.6% of the respondents felt that they had weak support from colleagues and disclosed negative work-related outcomes. Despite good colleague support, participants reported a relatively high score in the lack of institutional support (12.1%) and supervisor support (8.5%). About 4% admitted that professional efficacy was reduced, and 4.9% endured second-victim distress. However, 6.2% of the respondents reported an increase in their resilience after being involved in PSI.

Correlations among the variables are presented in Appendix A. Working experience and age had a notably high correlation (Pearson’s rho of 0.967 and a significant *p*-value) and led to omitting the working experience variable from further analysis.

As a prerequisite, before conducting a hierarchical linear regression, the related assumptions were tested and satisfied first. First, the sample size of 733 HCPs was deemed to be adequately consistent with the independent variables examined [89]. The assumption of singularity was also met, as there were no combined independent variables. Upon screening for correlations, no variable was highly correlated, except for age and working experience variables (supported by VIF and tolerance value), which led to the subsequent omission of working experience from later analysis. A check of multivariate outliers used Mahalanobis and Cook’s distance, which detected no outliers. Lastly, residual and scatter plots indicated that the assumptions of normality, linearity, and homoscedasticity were all satisfied [81].

As there were two interested outcomes, a three-stage hierarchical linear regression was first conducted with negative work-related outcomes as the dependent variable. The first block entered was the socio-demographic variables (age, gender, occupation, marital status, and last PSI encountered). The second block consisted of second-victim distress and professional efficacy, and the third block introduced colleagues, supervisors, and institutional support.

In the first block, the sociodemographic variables agreed for 3.8% of the variation in negative work-related outcomes and significantly contributed to the regression model, F (4726) = 7.1, *p* < 0.01). None of the sociodemographic variables contributed to the regression. After introducing the second block (second-victim distress and professional efficacy), an additional 64.6% of variation in negative work-related outcomes produced and concluded significant R^2^ change, F (2724) = 260.7, *p* < 0.01. Again, the sociodemographic variables did not contribute to the regression, as second-victim distress (β = 0.368, *p* < 0.001) and professional efficacy (β = 0.518, *p* < 0.001) created a significant contribution. The third block (colleague, supervisor, and institutional support) raised another additional 8% into the regression, and the R^2^ change was also significant F (9721) = 179.4, *p* < 0.001). Professional efficacy showed a higher beta value (β = 0.484, *p* < 0.001) than second-victim distress (β = 0.307, *p* < 0.001). All the types of support—colleague (β = 0.111, *p* < 0.001), supervisor (β = 0.064, *p* < 0.05), and institutional support (β = −0.065, *p* < 0.05)—were also significant and contributed to the regression. Sociodemographic variables remained insignificant. Table 2 explains the thorough block-by-block analysis.

As with the hierarchical linear regression of negative work-related outcomes, the difference was only the dependent variable: resilience. The block-by-block insertion replicated a similar process.

In the first block, the sociodemographic variables did not contribute to the regression model, F (4726) = 1.21, *p* > 0.05. After introducing the second block (second-victim distress and professional efficacy), an additional 4% of variation in resilience produced and concluded significant R^2^ change, F (6724) = 5.9, *p* < 0.001. Only second-victim distress (β = −0.22, *p* < 0.001) was significant in the regression. The final third block (colleague, supervisor, and institutional support) raised an additional 28.5% into the regression, and the R^2^ change was also significant F (9721) = 39.81, *p* < 0.001. The second-victim distress remained significant (β = −0.3, *p* < 0.001), and professional efficacy was also significant (β = −0.136, *p* < 0.001). The support continuum was all contributing to the regression: colleague (β = 0.34, *p* < 0.001), supervisor (β = 0.257, *p* < 0.001), and institutional support (β = 0.254, *p* < 0.001). Furthermore, not married—relative to married—also explained the slim significant value (β = 0.065, *p* < 0.05). Table 3 presents a thorough block-by-block analysis of the hierarchical regression model of resilience.

Four parallel multiple mediator models were developed. The first and second models used resilience as the outcome and professional efficacy (first model) and the second victim distress (second model) as the predictor. The third and fourth models provided negative work-related outcomes as the outcome, and second-victim distress (third model) and professional efficacy (the fourth model) as the predictor. All models shared the same mediators: colleague, supervisor, and institutional support. Please refer to the supplementary file for further elaboration.

## 4. Discussion

The highest mean recorded was for institutional and supervisor support. Unexpectedly, the level of second-victim distress was small and did not reveal the same pattern observed in other settings. Despite the difference, the low reduced professional efficacy level was in agreement with previously reported results [22,39,71,75,90]. Furthermore, in this study, the HCPs perceived a lack of institutional and supervisor support offered to them, contradicting the findings of previous studies [70,73,90,91]. Despite the non-agreement, findings in an Iranian healthcare setting concurred and substantiated that second victims were considered a novel notion in Iranian healthcare, and thus the reason for expecting inadequate support [74]. Concerning the negative work-related outcomes comprising turnover intention and absenteeism post-PSI, the low agreement, especially for absenteeism, was in accordance with situations in Denmark, Iran, Argentina, and the seminal studies by Burlison and Winning [22,39,71,74,75].

Across the globe, the healthcare system among countries is unique and, interestingly, the probable reason for the mixed reaction of negative work-related outcomes. In Argentina, as an instance of the bright side, the government offers financial compensation for full attendance and negotiates the luxury of mental health day if any workers report feeling any type of stress [71]. The turnover rate is also low in the country, possibly due to the difficulty of taking time off the high workload, heavy clinical burden, staffing shortage, and, perhaps, the additional constraints of the COVID-19 pandemic [73]. Malaysian healthcare settings were also stretched to their fullest; witnessing HCPs battled the pandemic with insurmountable stress and burnout, and coped with the constraint of quarantined staff [92,93]. As expected, the coping mechanism was extensively employed to ensure that the affected HCPs survived and thrived [27,94].

Hierarchical linear regression predicted the relationship between the predictors and the two outcomes: negative work-related outcomes and resilience. In fulfilling the prerequisite conditions, a significantly high correlation was detected between age and duration of working experience and was perhaps easily understood as the linear trajectory between them. Therefore, the duration of working experience was omitted from the later analysis.

Regression on negative work-related outcomes were predominantly contributed by second-victim distress and professional efficacy, with slight addition from the support triad. The literature explains that second-victim distress (psychological or physical distress) and reduced professional efficacy hugely affect negative work-related outcomes (turnover intention and absenteeism) [22,95,96,97]. The results confirmed previous similar findings in Iran [98], Lithuania [99], China [95], Singapore [100], and the United States of America [101].

In contrast to the first regression, the regression on resilience was remarkably decided by the support triad. However, second-victim distress and reduced professional efficacy only made trivial contributions toward resilience. Together, less stress and better mental health were emphasized to cultivate resilience [42]. Instead, the support triad became the biggest component of resilience. It is worth noting that the support triad, especially colleague support, posted a positive influence on resilience [102,103,104,105,106]. Apart from that, the sociodemographic status (age, duration from the last PSI encountered, marital status, position, and gender) did not carry any significance to either regression. However, single status was marginally significant but did not elicit much difference from the married status. Despite studies that favored females, married persons, and younger ages [107,108,109], most studies corroborated that there was no gender, age, or marital status association with being resilient [103,110,111,112,113,114]. Most importantly, the nature of individual job demands and job resources contributed the most decisive points for resilience [115].

Crucially, the support triad exhibited a detrimental mediating role in the pathway of second-victim distress and professional efficacy on negative work-related outcomes and resilience as verified by the multiple mediators (colleague, supervisor, and institutional support) model of the current study. In the pathway of second-victim distress and negative work-related outcomes, colleague and supervisor support were partially mediated (contemplative), whereas, upon using professional efficacy as the predictor, only colleague support remained. Institutional support did not react as a mediator. Although the magnitude was relatively small, together with second-victim distress and professional efficacy as respective predictors, colleague and supervisor support raised the magnitude of negative work-related outcomes, instead of supposedly ameliorating the effect. The aforementioned high score of perceived inadequate supervisor and institutional support conformed to this.

Further, this finding refuted previous studies, as colleague support was undoubtedly the most conducive and successful method to navigate through moments of embitterment [58,59,116]. Supervisor support was usually selected as the standard reference person and professional approval for subordinates [3,61]. The institutional support finding was considered insignificant, assuming the absence of top-down support initiatives or available initiatives disseminated with unsystematic, unstructured, and deviated approaches [18,37,117,118,119].

In comparison with safety-critical industries, such as emergency response (firefighters, police) [120,121], or the aviation industry [21], the support initiatives in healthcare lag behind by many years. In these critical fields, human factor contributions to error-prone records are carefully considered; with working hours strictly regulated, compromising safety procedures or standard operation procedures is never an option, and institutional responses of support are second to none [122,123].

However, in healthcare, the issue lingers around a punitive culture as the affected HCPs are afraid of blame, negative remarks, or being the object of stigmatization. In fact, systemic faults or the breakthrough of infinitesimal errors, as in Reason’s Swiss cheese model, are often overlooked [57,124,125]. Nevertheless, instilling a non-punitive culture would require a more welcoming atmosphere [27] and accommodate a more effective coping mechanism after experiencing PSI, which has been shown to halt the progress of second-victim distress or reduction in professional efficacy [126]. Effective coping mechanisms, as facilitated by the support triad, can be achieved by democratically discussing PSI on a neutral ground, and without judging remarks; this would result in healthcare reform or cultural transformation [4].

However, all three kinds of support in the pathway of second-victim distress and resilience were partially mediatory (competitive), and for the pathway to professional efficacy, only colleague support was affirmed. Upon the availability of adequate support, a decrease in the magnitude of second-victim distress and professional efficacy astonishingly boosted the mediated proportion of resilience to as high as 90%, with the largest share belonging to colleague support, similarly contributing to the regression model. Indeed, a similar atmosphere of inversed relation of support triad between distress and resilience was portrayed in other countries, such as the United States [127,128], China [129], and Australia [130]. As further evidence, a peer support program, the Resilience in Stressful Events (RISE) of John Hopkins [59], was evaluated for its effectiveness for second victims. As expected, RISE users reported greater resilience than non-users after facing PSI [131].

Accompanying the strengths of this study are its limitations. First, although this study attempted to recruit more doctors and assistant medical officers for a more diverse sample, the majority of the respondents were nurses. Perhaps the constraints of COVID-19 clinical care created a strain on the engagement of other professions in this study.

Due to the current pandemic, this study had to utilize online messaging platforms and e-mails to reach participants. Despite its feasibility, cost-effective procedure, and safety concerns of COVID-19, the online platform could not recognize interactive direct communication. Although complemented by a two-minute introductory video as assistance, some respondents found it difficult to answer the questionnaires.

As thoroughly discussed, the stigma of blame, judgment, or punitive culture in the Malaysian healthcare environment was a commanding challenge. Even though anonymity and confidentiality were the utmost concerns, possibly some respondents replied cautiously with a tendency of neutral remarks [132,133].

This was a cross-sectional study that had possibility of other confounding factors and limited power to analyze causality, but still provided a good description upon exploring second-victim circumstances in Malaysia. A further complex analysis, such as covariance-based structural equation modeling involving many latent constructs, longitudinal study, or causative multiple mediation model with other third variables such as moderation or covariates, could be exercised later to analyze more related variables (such as patient safety culture) [126,134,135]. Future studies could also choose a qualitative or mixed method approach that could perhaps better explain the suitable type of support. Then, the findings could be the foundation for creating support programs tailored to the local culture of Malaysian healthcare.

## 5. Conclusions

The triad of support—colleague, supervisor, and institutional support—demonstrated to be the panacea of the second victim’s repercussion, connecting the second-victim-related distress with negative work-related outcomes and the positive outcome of resilience. The challenge ahead is to inculcate good support practices, develop actionable working support systems, and shift the collective mind toward non-punitive healthcare.

## Figures and Tables

**Table 1:** Characteristics of respondents and domains (*n* = 733).

Characteristics and Domain (*n* = 733)	*n* (%)	Mean (SD)	Agreement (%)
**Gender**			
Male	103 (14.1%)		
Female	630 (85.9%)		
**Age (years)**		36.7 (7.7)	
**Race**			
Malay	712 (97.1%)		
Non-Malay	21 (2.9%)		
**Marital status**			
Married	618 (84.3%)		
Not married	115 (15.7%)		
**Current department**			
Anesthesiology and Critical Care	168 (22.3%)		
Internal Medicine	130 (17.7%)		
Pediatric	104(14.2%)		
Orthopedic	89 (12.1%)		
Obstetrics and Gynecology (O & G)	87 (11.9%)		
Surgery	79 (10.8%)		
Emergency and Trauma	30 (4.1%)		
Others	44 (6%)		
**Working experience (years)**		12 (7.7)	
**Last PSI encountered (years)**		2.4 (2.3)	
**Position**			
Nurses	596 (81.3%)		
Medical Officers	114 (15.6%)		
Assistant Medical Officers	23 (3.1%)		
**Domain**			
Second-victim distress		2.23 (1.02)	4.9
Professional efficacy		2.2 (1.0)	4.0
Colleague support		1.95(0.87)	1.6
Supervisor Support		2.86(0.95)	8.5
Institutional support		2.9(1.1)	12.1
Negative work-related outcomes		1.97(0.93)	4.0
Resilience		2.2(1.02)	6.2

SD: standard deviation.

**Table 2:** Hierarchical regression model of negative work-related outcomes.

	R	R^2^	R^2^ Change	β	SE	*T*
**Step 1**	0.194	0.038 ***				
Age				−0.051	0.018	−0.361
Last PSI encountered				0.015	0.015	0.414
Gender						
Male						
Female				−0.22	0.12	−1.798
Marital status						
Married						
Not married				−0.026	0.1	−0.695
Occupation				0.259	0.147	3.755
Nurses						
Medical officers				0.1	0.134	0.736
Assistant medical officers				0.08	0.22	0.351
**Step 2**	0.827	0.684 ***	0.646 ***			
Age				−0.034	0.01	−0.420
Last PSI encountered				−0.012	0.008	−0.548
Gender						
Male						
Female				−0.015	0.068	−1.674
Marital status						
Married						
Not married				−0.039	0.057	−1.78
Occupation						
Nurses						
Medical officers				−0.19	−0.077	−2.45
Assistant medical officers				0.079	0.127	0.625
Second-victim distress				0.368 ***	0.029	11.764
Professional efficacy				0.518 ***	0.030	16.494
**Step 3**	0.831	0.691 ***	0.08 *			
Age				−0.042	0.01	−0.517
Last PSI encountered				−0.012	0.008	−0.581
Gender						
Male						
Female				−0.095	0.07	−1.387
Marital status						
Married						
Not married				−0.033	0.057	−1.543
Occupation						
Nurses						
Medical officers				−0.126	0.08	−1.59
Assistant medical officers				0.11	0.126	0.842
Second-victim distress				0.307 ***	0.035	8.243
Professional efficacy				0.484 ***	0.031	14.93
Colleague support				0.111 ***	0.037	3.27
Supervisor support				0.064 *	0.028	2.32
Institutional support				−0.065 *	0.025	−2.376

Note: Statistical significance: * *p* < 0.05, *** *p* < 0.001, R^2^ = amount of variance explained by predictors, R^2^ change: additional variance in outcome variable, B = unstandardized coefficient, β = standardized coefficient, SE = standard error, t = estimated coefficient (B) divided by own SE.

**Table 3:** Hierarchical regression model of resilience.

	R	R^2^	R^2^ Change	β	SE	*T*
**Step 1**	0.081	0.007				
Age				−0.056	0.019	−0.385
Last PSI encountered				−0.048	0.016	−1.28
Gender						
Male						
Female				−0.08	0.12	−0.6
Marital status						
Married						
Not married				0.063	0.108	1.646
Occupation						
Nurses						
Medical officers				−0.28	0.146	−1.93
Assistant medical officers				0.3	0.24	1.241
**Step 2**	0.216	0.046 ***	0.04 *			
Age				−0.07	0.019	−0.5
Last PSI encountered				−0.036	0.016	−0.969
Gender						
Male						
Female				−0.076	0.13	−0.6
Marital status						
Married						
Not married				0.061	0.106	1.627
Occupation						
Nurses						
Medical officers				−0.2	0.146	−1.336
Assistant medical officers				0.283	0.237	1.197
Second-victim distress				−0.22 ***	0.054	−4.04
Professional efficacy				0.021	0.056	0.389
**Step 3**	0.576	0.332 ***	0.285 ***			
Age				−0.073	0.016	−0.61
Last PSI encountered						
Gender						
Male						
Female				0.05	0.11	0.436
Marital status						
Married						
Not married				0.065 *	0.09	2.039
Occupation						
Nurses						
Medical officers				−0.132	0.13	−1.053
Assistant medical officers				0.35	0.2	1.742
Second-victim distress				−0.3 ***	0.055	−5.36
Professional efficacy				−0.136 ***	0.05	−2.858
Colleague support				0.34 ***	0.06	6.79
Supervisor support				0.257 ***	0.044	6.373
Institutional support				0.254 ***	0.04	6.3

Note: Statistical significance: * *p* < 0.05, *** *p* < 0.001, R^2^ = amount of variance explained by predictors, R^2^ change: additional variance in outcome variable, B = unstandardized coefficient, β = standardized coefficient, SE = standard error, t = estimated coefficient (B) divided by own SE.

## Data Availability

The data are not publicly available due to privacy and confidentiality. However, restrictions apply to the availability of hospital data and are available from the authors with the permission of the organization.

## Supplementary Materials

The following supporting information can be downloaded at: https://www.mdpi.com/article/10.3390/ijerph19116454/s1, Figure S1a: Multiple mediation model of the relationship between professional efficacy and resilience mediated through colleague, supervisor, and institutional support; Figure S1b: Multiple mediation model of the relationship between second victim distress and resilience mediated through colleague, supervisor, and institutional support; Figure S1c: Multiple mediation model of the relationship between second victim distress and negative work-related outcomes mediated through colleague, supervisor, and institutional support; Figure S1d: Multiple mediation model of the relationship between professional efficacy and negative work-related outcomes mediated through colleague, supervisor, and institutional support; Table S1: Specific direct effects, specific indirect effects, and total indirect effects of professional efficacy on resilience; Table S2: Specific direct effects, specific indirect effects, and total indirect effects of second victim distress (SVD) on resilience; Table S3: Specific direct effects, specific indirect effects, and total indirect effects of second victim distress (SVD) on negative work-related outcomes; Table S4: Specific direct effects, specific indirect effects, and total indirect effects of professional efficacy on negative work-related outcomes.

## Appendix A

**Table A1:** Correlation of all continuous variables (*n* = 733).

Variables	Age	WE	LP	SVD	PEF	CS	SS	IS	NWRO	Resi
Age	1.000 ***	0.967 ***	0.251 ***	−0.286 ***	−0.284 ***	−0.215 ***	0.032	−0.069	−0.225 ***	0.022
WE	0.967 ***	1.000	0.204 ***	−0.210 ***	−0.231 ***	−0.218 ***	0.020	−0.027	−0.217 ***	0.027
LP	0.251	0.204 ***	1.000	0.046	−0.021	−0.005	−0.097	−0.069	0.018	−0.081
SVD	−0.286 ***	−0.210 ***	0.046	1.000 ***	0.741 ***	0.764 ***	−0.172 ***	−0.119	0.75 ***	−0.124 **
PEF	−0.284 ***	−0.231 ***	−0.021	0.741 ***	1.000	0.716 ***	−0.112 *	−0.037	0.771 ***	−0.069
CS	−0.215 ***	−0.218 ***	−0.005	0.764 ***	0.716 ***	1.000	−0.055	0.020	0.713 ***	−0.026
SS	−0.011	−0.015	−0.097	−0.172 ***	−0.112	−0.055	1.000	0.645	−0.123 **	0.546 ***
IS	−0.069 *	−0.069 *	−0.059	−0.039	0.009	−0.010	0.677 ***	1.000	−0.052	0.472 ***
NWRO	−0.225 ***	−0.217 ***	0.018	0.75 ***	0.771 ***	0.713 ***	−0.123	−0.052	1.000	−0.111 ***
Resi	0.337	0.299	0.057	0.008	0.088	0.304	0.000	0.000	−0.111	1.000

Note: Statistical significance: * *p* < 0.05, ** *p* < 0.01, *** *p* < 0.001, WE (working experience), Last PSI encountered (LP), Second-victim distress (SVD), Professional efficacy (PEF), Colleague support (CS), Supervisor support (SS), Institutional support (IS), Negative work-related outcomes (NWRO), Resilience (R).
