# Supplementary material for: The Effect of Second-Victim-Related Distress and Support on Work-Related Outcomes in Tertiary Care Hospitals in Kelantan, Malaysia
Source: Int J Environ Res Public Health. 2022 May 26;19(11):6454. doi: 10.3390/ijerph19116454 (PMC9180130; doi:10.3390/ijerph19116454)
Supplement: Supplementary file 1 [file ijerph-19-06454-s001.zip › ijerph-1689552-supplementary.pdf]

Supplementary Document

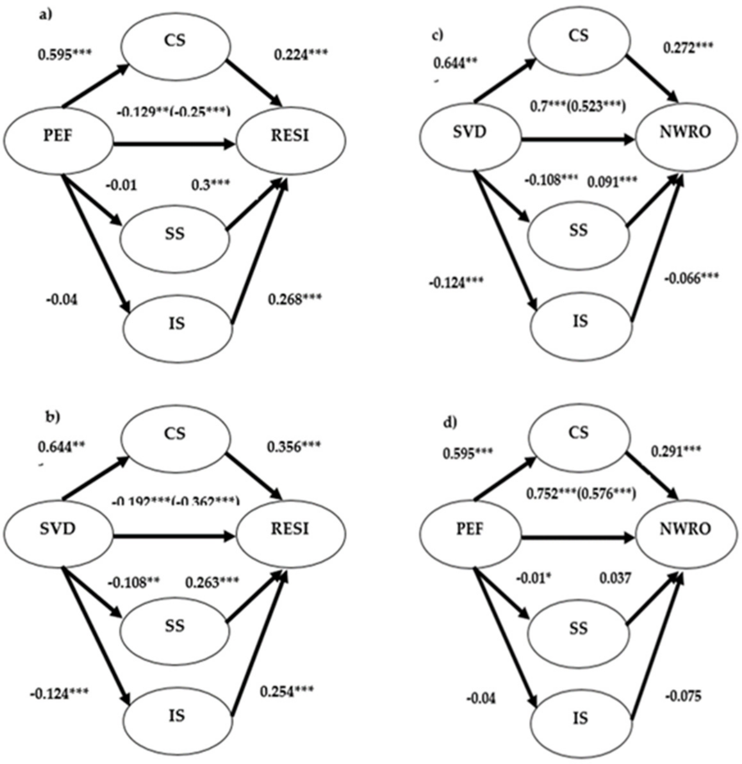

Figure S1a) Multiple mediation model of the relationship between professional efficacy and resilience mediated through colleague, supervisor, and institutional support.
Figure S1b) Multiple mediation model of the relationship between second victim distress and resilience mediated through colleague, supervisor, and institutional support.
Figure S1c) Multiple mediation model of the relationship between second victim distress and negative work-related outcomes mediated through colleague, supervisor, and institutional support.
Figure S1d) Multiple mediation model of the relationship between professional efficacy and negative work-related outcomes mediated through colleague, supervisor, and institutional support.
Note: Statistical significance: *p <0.05, **p <0.01, ***p <0.001, SVD: second victim distress, PEF: professional efficacy, CS: colleague support, SS: supervisor support, IS: institutional support, NWRO: negative work-related outcomes, RESI: resilience

As shown in Figure S1a and Table S1, the total effect and specific direct effect of professional efficacy on resilience was both significant (standardized estimate [Std. estimate] = -0.129, p < 0.001 and Std. estimate = -0.25, p <0.001). Professional efficacy was also related to resilience through colleague support (Std. estimate = 0.133, p <0.001) and showed significance with the total indirect effect (Std. estimate = 0.12, p <0.001). Overall, colleague support, which accounted for 93% (0.12/-0.129) [Std. estimate of total indirect effect/Std. estimate of total effect), was considered partially and competitive in the pathway from professional efficacy and resilience.

Table S1: Specific direct effects, specific indirect effects, and total indirect effects of professional efficacy on resilience**.**

			95% CI of estimates (after 5000 bootstraps)	
	Estimates	SE	Lower	Upper
Specific direct effect				
PEF → Resi	-0.25***	-0.044	-0.344	-0.163
PEF → CS	0.595***	0.023	0.549	0.641

PEF → SS	-0.01	0.035	-0.078	0.059
PEF → IS	-0.04	0.039	-0.117	0.038
CS→ Resi	0.356***	0.057	0.245	0.467
SS→ Resi	0.263***	0.043	0.178	0.348
IS→ Resi	0.268***	0.039	0.191	0.344
Specific indirect effects				
PEF→CS→ Resi	0.133***	0.03	0.074	0.193
PEF →SS→ Resi	-0.003	0.01	-0.024	0.018
PEF →IS→Resi	-0.011	0.011	-0.031	0.01
Total indirect	0.12***	0.036	0.049	0.19
Total effect	-0.129***	0.037	-0.202	-0.056

Note: Statistical significance: *p <0.05, **p <0.01, ***p <0.001.

Figure S1b and Table S2 demonstrated the total effect and specific direct effect of second victim distress on resilience that were related significantly (Std. estimate = -0.192, p <0.001 and Std. estimate = -0.362, p <0.001. Other specific direct effects were also found in significant relationships. In the pathway of second victim distress and resilience, all three specific indirect effects were all significant: colleague (Std. estimate = 0.229, p <0.001), supervisor (Std. estimate = -0.028, p <0.001), and institutional support (Std. estimate = -0.031, p <0.001), and agreed with the significant total indirect effect (Std. estimate = 0.17, p <0.001). In summary, the supports accounted for 88.5% (0.17/|-0.192|) and was partially and competitive in the pathway of second victim distress and resilience.

Table S2. Specific direct effects, specific indirect effects, and total indirect effects of second victim distress (SVD) on resilience.

			95% CI of estimates (after 5000 bootstraps)	
	Estimates	SE	Lower	Upper
Specific direct effects				
SVD → Resi	-0.362***	0.048	-0.457	-0.267
SVD→ CS	0.644***	0.02	0.605	0.684
SVD→ SS	-0.108***	0.034	-0.174	-0.042
SVD→ IS	-0.124***	0.038	-0.2	-0.05
CS→ Resi	0.356***	0.057	0.245	0.467
SS→ Resi	0.263***	0.043	0.178	0.348
IS→ Resi	0.268***	0.038	0.178	0.329
Specific indirect effects				
SVD→CS→ Resi	0.229***	0.037	0.156	0.303
SVD→SS→ Resi	-0.028***	0.01	-0.048	-0.009
SVD→IS→Resi	-0.031***	0.011	-0.052	-0.01
Total indirect effect	0.17***	0.042	0.087	0.253
Total effect	-0.192***	0.036	-0.263	-0.122

Note: Statistical significance: *p <0.05, **p <0.01, ***p <0.001.

The total and specific direct effects of second victim distress and negative work-related outcomes were both significantly related (Std. estimate = 0.7, p <0.001 and Std. estimate = 0.523, p <0.001) as explained by Figure S1c and Table S3. Off the same pathway, colleague support (Std. estimate = 0.175, p <0.001) and supervisor support (Std. estimate = -0.01, p <0.05) respectively proved the specific indirect effects and together contributed to the significant total indirect effect (Std. estimate = 0.174, p <0.001). Other specific direct effects were all significant. Both supports offered 24.9% of the explained variance and concluded as partial and contemplative in the relationship between second victim distress and negative work-related outcomes.

Table S3. Specific direct effects, specific indirect effects, and total indirect effects of second victim distress (SVD) on negative work-related outcomes.

	Estimates	SE	95% CI of estimates (after 5000 bootstraps)	
			Lower	Upper
Specific direct effect				
SVD → NWRO	0.523***	0.035	0.455	0.592
SVD→ CS	0.644***	0.02	-0.12	-0.011
SVD→ SS	-0.108***	0.034	-0.174	-0.042
SVD→ IS	-0.124***	0.038	-0.2	-0.05
CS→ NWRO	0.272***	0.041	0.192	0.352
SS→ NWRO	0.091***	0.031	0.029	0.152
IS→ NWRO	-0.066*	0.028	-0.12	-0.011
Specific indirect effects				
SVD→CS→ NWRO	0.175***	0.027	0.122	0.228
SVD→SS→ NWRO	-0.01*	0.005	-0.019	-0.001
SVD→IS→NWRO	0.008	0.004	0.000	0.016
Total indirect	0.174***	0.028	0.119	0.228
Total effect	0.7***	0.023	0.653	0.742

Note: Statistical significance: *p <0.05, **p <0.01, ***p <0.001.

Figure S1d and Table S4 explain the relationship between professional efficacy and negative work-related outcomes in the presence of multiple mediators: colleague, supervisor, and institutional support. The total and specific direct effect of professional efficacy and negative work-related outcomes were both significant (Std. estimate = 0.752, p <0.001 and Std. estimate = 0.576, p <0.001). Only colleague support proved significant for the specific indirect effect (Std. estimate = 0.173, p <0.001), resulted in a significant total indirect effect (Std. estimate = 0.173, p <0.001), and summarized for 23.4% of the professional efficacy on negative work-related outcomes. In this regard, this was partial and contemplative.

Table S4. Specific direct effects, specific indirect effects, and total indirect effects of professional efficacy on negative work-related outcomes.

	Estimates	SE	95% CI of estimates (after 5000 bootstraps)	
			Lower	Upper
Specific direct effect				
PEF → NWRO	0.576***	0.028	0.52	0.631
PEF→ CS	0.595***	0.023	0.549	0.641
PEF→ SS	-0.01	0.035	-0.078	0.038
PEF→ IS	-0.04	0.039	-0.117	0.038
CS→ NWRO	0.291***	0.033	0.227	0.355
SS→ NWRO	0.091***	0.031	0.029	0.152
IS→ NWRO	-0.066***	0.028	-0.12	-0.011
Specific indirect effects				
PEF→CS→ NWRO	0.173***	0.021	0.133	0.214
PEF→SS→ NWRO	0.000	0.001	-0.003	0.002
PEF→IS→NWRO	0.003	0.003	-0.003	0.009
Total indirect	0.176***	0.021	0.135	0.217
Total effect	0.752***	0.022	0.709	0.794

Note: Statistical significance: *p <0.05, **p <0.01, ***p <0.001.
